# Applicability of RPMI 2650 and Calu-3 Cell Models for Evaluation of Nasal Formulations

**DOI:** 10.3390/pharmaceutics14020369

**Published:** 2022-02-06

**Authors:** Nadica Sibinovska, Simon Žakelj, Jurij Trontelj, Katja Kristan

**Affiliations:** 1 Department of Biopharmaceutics and Pharmacokinetics, Faculty of Pharmacy, University of Ljubljana, Aškerčeva Cesta 7, 1000 Ljubljana, Slovenia; sibinovska.nadica@gmail.com (N.S.); Simon.Zakelj@ffa.uni-lj.si (S.Ž.); Jurij.Trontelj@ffa.uni-lj.si (J.T.); 2 Faculty of Medicine, Institute of Biochemistry and Molecular Genetics, University of Ljubljana, Vrazov trg 2, 1000 Ljubljana, Slovenia; 3 Sandoz Development Center Slovenia, Lek Pharmaceuticals, d.d., Verovškova 57, 1526 Ljubljana, Slovenia

**Keywords:** RPMI 2650 cell line, Calu-3 cell line, nasal drug formulations, nasal epithelium, drug permeability

## Abstract

The RPMI 2650 and Calu-3 cell lines have been previously evaluated as models of the nasal and airway epithelial barrier, and they have demonstrated the potential to be used in drug permeation studies. However, limited data exist on the utilization of these two cell models for the assessment of nasal formulations. In our study, we tested these cell lines for the evaluation of in vitro permeation of intranasally administered drugs having a local and systemic effect from different solution- and suspension-based formulations to observe how the effects of formulations reflect on the measured in vitro drug permeability. Both models were shown to be sufficiently discriminative and able to reveal the effect of formulation compositions on drug permeability, as they demonstrated differences in the in vitro drug permeation comparable to the in vivo bioavailability. Good correlation with the available bioavailability data was also established for a limited number of drugs formulated as intranasal solutions. The investigated cell lines can be applied to the evaluation of in vitro permeation of intranasally administered drugs with a local and systemic effect from solution- and suspension-based formulations.

## 1. Introduction

When drugs are administered nasally to achieve a local or a systemic effect, it is difficult to clinically observe their interaction with the nasal mucosa in terms of uptake into or diffusion through the epithelium. Performing permeability studies is necessary to predict nasal drug absorption and elucidate the transport mechanisms through the nasal epithelial barrier. In vitro models of various complexities [1,2,3,4,5] and in situ models [6] are being established for this purpose. The RPMI 2650 and Calu-3 cell lines are among the simplest models applicable, and we have previously demonstrated that these two cell lines grown at an air–liquid (A–L) interface are highly useful for distinguishing the permeability of low or moderately permeable compounds dissolved in pure balanced salt solution from the permeability of those with high permeability designation [7,8].

While the biopharmaceutical classification can be convenient to estimate the systemic absorption, which can indicate efficacy, or the undesired systemic exposure indicating safety of an intranasally administered drug, in vitro models of the nasal mucosa are also needed for formulation development of novel and generic drugs. The analogy of utilizing the biopharmaceutical classification system (BCS) with the gastrointestinal absorption, which is very helpful with the permeability classification of orally administered drugs, is not truly applicable to the intranasally administered formulations, since the dilution of the formulation on the nasal mucosa is much lower compared to the intestinal situation. The intranasal formulation may be intended to prolong the retention of a drug on the mucosal surface through mucoadhesion or modification of the rheology of mucus, to moisturize the dry nasal mucosa, or to enhance the drug dissolution or permeation across the nasal epithelial barrier [9]. These functions can be designed into the formulations through the optimization of composition, and the choice of the excipients can influence the drug permeability.

Some locally acting drugs (xylometazoline, oxymetazoline, naphazoline, azelastine, ipratropium, triamcinolone acetonide, betamethasone sodium phosphate) and drugs intended for nose-to-brain delivery (sumatriptan, zolmitriptan) can be incorporated in solution-based formulations, while some of the locally acting intranasally administered drugs (e.g., corticosteroids) are formulated as suspensions, enabling prolonged drug release. Such formulations, however, are especially inconvenient for studying and comparing from the perspective of drug permeability and pose a challenge in terms of establishing a proper experimental setup, as well as reporting the results. In general, it is reasonable to present the results of the permeability assays as flux values, besides the more commonly reported apparent permeability coefficient (P_app_), due to the exact donor concentration at the surface of the permeability barrier being unknown, as in the case of suspensions or aerosols [10].

The RPMI 2650 and Calu-3 cell lines have been the subject of investigation as models of the nasal epithelial barrier [5,7,11,12,13,14,15,16,17]. These cell lines differ in terms of origin of the cells (nasal origin of RPMI 2650 vs. bronchial of Calu-3), the TEER (low TEER values are normal for RPMI 2650, which is a leaky type of epithelium) and formation of monolayers (Calu-3) or multilayers (RPMI 2650) [12,18]. The two cell models have been utilized by several research groups [19,20,21,22,23,24] for nasal drug permeability assessment. However, to the best of our knowledge, an attempt to investigate the applicability of these two cell models of the nasal epithelial barrier for the evaluation of in vitro permeation of a large number of intranasally administered drugs from different marketed formulations has not been made yet.

The central purpose of this study was to evaluate and compare the applicability of the RPMI 2650 and Calu-3 cell models of the nasal epithelial barrier for permeability evaluation of intranasal drug formulations, by investigating whether the cell-based models can detect the influence of formulations composition (e.g., different excipients used, presence of another drug, different pH) on the permeability of the active drug. Additionally, the work presented here aims to elucidate the impact of the formulations on the cell layer integrity and viability throughout the experiments. Therefore, in order to evaluate the usefulness of RPMI 2650 and Calu-3 cell layers for drug permeability measurements in the presence of complete formulations, as well as to indicate some important aspects of intranasal formulation testing, we used a wide variety of drugs incorporated into different commercially available intranasal formulations. Some aspects of the nasal formulation permeability testing which have been highlighted previously are also included in our present work. Namely, when drug formulations are administered intranasally, physiological dilution by the nasal mucus occurs and a 10-fold formulation dilution prior to testing is considered as a clinically relevant dilution factor in some studies [25,26]. Therefore, the permeability of the intranasally administered drugs incorporated into marketed formulations was tested without any prior dilution, as well as with the clinically relevant 10-fold formulation dilution prior to testing. The permeability of intranasal drugs was also investigated by preparing pure solutions of the drugs in balanced salt solution (pH 7.4), where applicable. Moreover, increasingly popular drug combinations in a single formulation were tested. Another feature of the applicability of the RPMI 2650 and Calu-3 cell models for permeability testing of low-permeable drugs was examined in terms of practicality regarding the analysis of permeated drug amount. Whenever possible, the demonstrated in vitro differences in drug permeation between formulations were compared to the available in vivo data.

## 2. Materials and Methods

### 2.1. Materials

Advanced Minimum Essential medium (A-MEM), heat-inactivated fetal bovine serum (FBS), GlutaMAX™ Supplement, TrypLE™ Select, Hank’s balanced salt solution (HBSS), and 4-(2-hydroxyethyl)piperazine-1-ethanesulfonic acid (HEPES) were purchased from Thermo Fisher Scientific (Waltham, MA, USA). Tissue culture flasks were obtained from Sarstedt (Nümbrecht, Germany), and Millicell^®^ 24-well receiver trays and 24-well cell culture plate assemblies (24-well cell culture plate, single-well feeder tray and lid) were purchased from Merck Millipore (Tullagreen, Ireland). The chemicals used for mobile phase preparation were obtained from Sigma-Aldrich and J.T. Baker/Avantor Performance Materials (Radnor, PA, USA), and the investigated drugs from Sigma-Aldrich (Munich, Germany). For the preparation of solutions of sumatriptan and ipratropium, an assay buffer composed of HBSS with 0.01M HEPES was utilized, while assay buffer with max. 1% methanol (MeOH) or dimethyl sulfoxide (DMSO) (Sigma-Aldrich (Munich, Germany)) was used for preparation of solutions of azelastine, or zolmitriptan, budesonide and triamcinolone acetonide, respectively. For the permeability assays of the nasal formulations, an assay buffer was used for all nasal preparations, except for the second-generation intranasal corticosteroids and the first-generation corticosteroid beclomethasone dipropionate. When second-generation intranasal corticosteroid and beclomethasone dipropionate formulations were evaluated, an assay buffer with 4% BSA (*w/v*) (Sigma-Aldrich (Munich, Germany)) was used.

### 2.2. Cell Cultures

RPMI 2650 cells were purchased from the European Collection of Cell Cultures, Health Protection Agency at passage number 10 (Cat.No. 88031602, Lot No. 10D028, STR verification: 08 June 2010, PCR-based mycoplasma detection: 11 June 2010). They were used for permeability assays between passages 31–34. Calu-3 cells were from the American Type Culture Collection (LGC Standards, Cat No. ATCC-HTB-55). Lot No. 70009867, passage number 17 was used from passages 22–32 and Lot No. 58052345, passage number 18, from passages 53 to 58. STR verification and tests for mycoplasma contamination (Hoechst DNA stain, agar culture and PCR-based assay) were performed. Both cell lines were cultured in tissue culture flasks from polystyrene, kept at 37 °C, in >95% humidified atmosphere of air with 5% CO_2_. The growth medium was A-MEM supplemented with 2% GlutaMAX™ and 2.5% FBS. It was replenished every 48 h (except for the weekends). When 80–90% confluence in the culture flasks was reached, the cells were harvested by TrypLE^™^Select and seeded on polyethylene terephthalate (PET) membranes (1 µm pores, 0.7 cm^2^) at a density of 2 × 10^5^ cells/cm² in Millicell^®^ 24-well cell culture plates. When seeding the cells, 400 µL medium/insert of cell growth medium was added to the apical and 22–24 mL / feeder tray to the basolateral side. The A–L interface was established by removing the medium from the apical compartment one day post-seeding. The cell culture medium was changed every 2–3 days. The cells were used for permeability assays after three weeks of culturing.

### 2.3. Permeability Assays

#### 2.3.1. Permeability Assays of Formulations Containing Intranasal Corticosteroids with Low Aqueous Solubility (Beclomethasone Dipropionate, Fluticasone Propionate, Mometasone Furoate, Ciclesonide)

The RPMI 2650 and Calu-3 cells were cultured as previously described [7,8]. Prior to conducting the permeability assay, the cell layers were rinsed twice with assay buffer, pre-warmed at 37 °C, followed by TEER measurement (REMS autosampler, World Precision Instruments, Sarasota, USA and EVOM Epithelial Voltohmmeter with STX2 chopstick electrodes, World Precision Instruments, Sarasota, FL, USA). Only RPMI 2650 or Calu-3 cells with TEER values > 30 Ωcm^2^ or >275 Ωcm^2^, respectively, measured on the day of the experiment were considered suitable to be utilized in permeability assays [7,8].

The composition of all tested nasal formulations and in-house prepared solutions of different active substances are shown in the Supplementary Materials (Table S1). All transport studies were conducted in apical to basolateral (A-B) direction. A measure of 100 μL of undiluted and 10-fold diluted nasal formulations (dilution by an isotonic assay buffer) were added to the apical side, while 800 μL of pre-warmed assay buffer with 4% BSA were added to the basolateral compartment. The 24-well cell culture plates with RPMI 2650/Calu-3 cells were incubated at 37 °C in a humidified 5%CO_2_/95% air atmosphere throughout the permeability assays. The 100 μL (for undiluted formulations) and 150 µL (for the 10-fold diluted formulations) samples were withdrawn from the acceptor wells at predetermined time points (30, 60, 90, 120, 180 and 240 min). The withdrawn volume was replaced with the appropriate fresh assay buffer with 4% BSA. At the end of the permeability assay, 20 µL samples of the tested formulations were withdrawn from the apical compartment and added into microcentrifuge tubes containing 980 µL ice-cold 60% MeOH, in order to determine the remaining amount of corticosteroids in the nasal formulations on the donor side of the cells.

Following sample collection, the cells were gently washed with assay buffer without BSA and the TEER was measured. RPMI 2650 or Calu-3 cells with TEER values > 25 Ωcm^2^ and >250 Ωcm^2^, respectively, at the end of the permeability assays with the different nasal formulations were considered to have maintained barrier integrity [7,8]. These two criteria, as well as the criterion on adequate cell viability by performing an LDH cytotoxicity assay (see Section 2.4), were taken into account when deciding on inclusion/exclusion of the obtained results from the permeability assays into calculation of the apparent permeability coefficient (P_app_) or flux values, alongside the main criterion of observing linearity in the plot of the amount or mass of the investigated drug permeated to the acceptor side versus time. After measuring TEER, the membranes with cells were removed from the 24-well cell culture plates in order to determine the mass balance, and each individual membrane was placed in a microcentrifuge tube with 600 µL of ultrapure water. The microcentrifuge tubes were further stored at −20 °C until cell lysis. Frozen cells were thawed and sonicated (60 s, 40 mA), and then 800 µL ice-cold acidified MeOH (0.1% HCOOH in MeOH) was added to each 200 µL sample and left at −20 °C for at least 24 h to allow protein precipitation. After that, the samples were centrifuged at 15,000× *g* for 10 min at 4 °C. The supernatants were transferred onto Agilent polypropylene round bottom 96-well plates and subjected to LC-MS/MS analysis. In order to determine the mass balance, each individual well on the Millicell 24-well collection tray (Cat. no. PSMW010R5, Millipore) was rinsed twice with 800 µL 60% MeOH. A total volume of 1600 µL was collected in Eppendorf tubes and stored at −20 °C for at least 24 h to allow precipitation of any remaining proteins in the wells. The samples were centrifuged at 15,000× *g* for 10 min at 4 °C and the supernatants were then subjected to LC-MS/MS analysis. The amount of each investigated corticosteroid in the samples was calculated from a calibration curve (Section 2.6 Analytical methods), generated by spiking assay buffer with 4% BSA with adequate volumes of dissolved active substance in MeOH, followed by protein precipitation with acidified MeOH at a ratio of 1:4 (calibration point:acidified MeOH).

#### 2.3.2. Permeability Assays for Nasal Formulations of First-Generation Intranasal Corticosteroids with Higher Aqueous Solubility and Other Nasally Administered Drugs

The permeability studies for the evaluated nasal formulations of the first-generation corticosteroids having higher aqueous solubility (triamcinolone acetonide and budesonide) and all other nasally administered drugs were carried out in a similar manner as described in Section 2.3.1. Pre-warmed assay buffer without BSA was used throughout the experiments. The 100 μL samples were withdrawn from the acceptor wells at predetermined time points (30, 60, 90, 120 and 180 min). The withdrawn volume was replaced with the appropriate fresh assay buffer. The permeability assay for the in-house prepared triamcinolone acetonide solution was conducted as described in our previous work [23]. At the end of the experiments, the cells were gently washed with assay buffer and the TEER was measured as previously described. The cell layer integrity was additionally evaluated for the nasally administered drugs, except for the corticosteroids, by incubating the cells with 20 μM Lucifer Yellow (LY) solution for 1 h under standard incubating conditions. Samples of 100 μL were withdrawn from the acceptor wells post-incubation and their fluorescence was measured using microtiter plate reader (Infinite M1000, Tecan, Switzerland). Only RPMI 2650 or Calu-3 cells having P_app_ of LY ≤ 1 × 10^−5^ cm/s and ≤1 × 10^−^^6^ cm/s or alternatively having TEER values > 25 Ωcm^2^ and >250 Ωcm^2^, respectively, at the end of the permeability assays with the different nasal formulations were deemed as having maintained the cell layer integrity [7,8].

### 2.4. LDH Cytotoxicity Assay

The in vitro cytotoxic effect of the tested undiluted and 10-fold diluted nasal solution-based formulations of xylometazoline, oxymetazoline, naphazoline, as well as azelastine, and suspension-based formulations of fluticasone propionate, ciclesonide, mometasone and budesonide was investigated by performing an LDH cytotoxicity assay (CyQUANT LDH Cytotoxicity Assay Kit, Invitrogen, ThermoFisher Scientific, Waltham, MA, USA), with a minor modification of the manufacturer’s instruction manual. The cell lines were grown on 24-well plates for 3 weeks, as described in Section 2.2. For obtaining samples for the maximum and spontaneous LDH activity of the two cell models, the cell culturing media was removed and assay buffer was placed on the basolateral side, while on the apical side, the cells were treated for 45 min with 10X Lysis Buffer diluted with assay buffer (1:10) (maximum LDH activity) and with sterile ultrapure water diluted with assay buffer (1:10) (spontaneous LDH activity). After 45 min incubation at 37 °C, 50 µL of samples were withdrawn from the basolateral side. The samples from the cells treated with nasal formulations were obtained by collecting the remaining volume of samples after the last sampling time point at the end of the permeability assays and were stored at −20 °C until further analysis. Measures of 50 µL of samples were transferred to a 96-well flat-bottom plate, and 50 µL of a reaction mixture was added to each sample well. The 96-well flat-bottom plate was kept at room temperature for 30 min, protected from light. After 30 min incubation 50 µL of stop solution was added to each well and the absorbance was measured at 490 nm and 680 nm. In order to determine the LDH activity, the absorbance value at 680 nm (background) was subtracted from the absorbance at 490 nm, and the % cytotoxicity was calculated according to the following Equation (1):

(1) $$\%\;cytotoxiciy=\frac{Compound-treated\;LDH\;activity-Spontaneous\;LDH\;activity}{Maximum\;LDH\;activity-Spontaneous\;LDH\;activity}\times 100$$

### 2.5. Osmolarity Measurement

The osmolarity of undiluted and 10-fold diluted marketed nasal formulations was measured using freeze-point osmometer Osmomat 3000 (Gonotec GmbH, Berlin, Germany). Measures of 50 µL of each undiluted formulation as well as of nasal formulations diluted 10-fold with the assay buffer were used for osmolarity measurement.

### 2.6. Analytical Methods

The quantitative analysis of all the investigated intranasally administered drugs, with the exception of the second-generation corticosteroids and the first-generation intranasal corticosteroid beclomethasone dipropionate (permeability assays with both cell lines) and ipratropium (permeability assays with RPMI 2650 only) was performed by ultra-high-pressure liquid chromatography (UHPLC Acquity separations module equipped with photodiode array detector, Empower software) (Waters, Milford, MA, USA). The UHPLC parameters are described in the Supplementary Materials (Table S2). Quantification of the second-generation corticosteroids and beclomethasone dipropionate (permeability assays with both cell lines) and ipratropium (permeability assays with Calu-3 cells) was performed by the LC-MS/MS method described below, and the MRM acquisition data used to quantify the analytes are presented in the Supplementary Materials (Table S3). The acceptor compartment samples were injected without any sample pretreatment, while the donor compartment samples were first centrifuged and then diluted 50–10,000 times to meet the linear calibration range of the LC-MS/MS method.

The sample analysis was performed on an Agilent liquid chromatograph 1290 Infinity I coupled to an Agilent 6460 triple quadrupole mass spectrometer equipped with a JetStream^®^ electrospray interface (Agilent Technologies, Santa Clara, CA, USA). The injection volume was 0.1 µL for diluted donor compartment samples and the cell-lysates, while for the acceptor compartment samples, the injection volume was 3 µL. Mobile phase A was MilliQ water (obtained by A10 Advantage Millipore MilliQ water purification system, TOC ≤ 3 ppb, Millipore Corp., Billerica, MA, USA) and the mobile phase B was 100% LC-MS grade methanol. The column Kinetex C18 50 × 2.1 mm, 2.6 mm particles (Phenomenex, Torrance, CA, USA) were thermostated at 40 °C and the elution was performed by the following gradient: (time (min), B (%), flow rate (mL/min)): (0 min, 55%, 0.4 mL/min), (3 min, 68%, 0.4 mL/min), (3.5 min, 95%, 0.4 mL/min), (5.4 min, 95%, 0.4 mL/min), (5.5 min, 55%, 0.6 mL/min), (6.3 min, 55%, 0.5 mL/min), (6.4 min, 55%, 0.4 mL/min). The total run-time was 6.5 min, including re-equilibration. The instrument was controlled and the MRM data was extracted and processed by MassHunter Workstation software B.06.00 (Agilent Technologies, Santa Clara, CA, USA) (Table S2). The method was calibrated in the matrix, matched with the study samples with the following final analyte concentrations: 0.13, 0.44, 1.6, 5.0, 20, 70, 230, 820, 2860, and 10000 ng/mL. The method was checked for accuracy (85–115%), precision (RSD < 15%), selectivity, and linear dynamic range with the determination of lower and upper limits of quantitation.

### 2.7. Permeability Data Analysis

The P_app_ values were calculated using the Equation (2):

(2) $$P_{\mathrm{app}}=k_{d}\times \frac{1}{A\times C_{0}}$$

where k_d_ (mol/s or mg/s) is the slope of the linear section in the amount or mass of investigated drug substance permeated to the acceptor side versus time plot, A is the exposed surface area of the cell monolayer (0.7 cm^2^), $C_{0}$ (M or mg/mL) is the average initial concentration of drug substance in donor wells.

Permeation rate (i.e., flux) calculation was performed by plotting the cumulative amount of drug permeated per unit of surface area against time. The slope of the regression line was calculated as the permeation rate (flux) (only linear part of the curve was used for calculation).

### 2.8. Statistical Analysis

The statistical comparisons were performed using GraphPadPrism version 8.1.2 for Windows (GraphPad Software, San Diego, CA, USA). The calculated P_app_ and flux values of the drugs incorporated in the tested formulations are expressed as mean ± standard deviation (SD) of at least three replicates. A two-tailed *t*-test was performed for the comparison of means for azelastine and fluticasone propionate. All analyses were conducted at a significance level of 0.05.

## 3. Results and Discussion

### 3.1. Permeability of Drugs from Solution-Based Nasal Formulations and Correlation with In Vivo Data

The permeability of drugs with a local (xylometazoline, oxymetazoline, naphazoline, azelastine, ipratropium) and systemic effect (sumatriptan, zolmitriptan) from solution-based formulations was tested across the RPMI 2650 and Calu-3 cell models, as undiluted formulations and formulations diluted 10-fold with an isotonic assay buffer, and the obtained P_app_ values are shown in Table 1. Moreover, the permeability of these drugs, as well of two first-generation corticosteroids (triamcinolone acetonide and budesonide) from solutions prepared in assay buffer (with 1% DMSO or 1% MeOH, when necessary) was investigated; the results are represented in Table 1.

Solution-based formulations of three nasal decongestant drugs with different drug concentrations or containing a combination of a decongestant with another drug have been tested. Namely, besides solution-based nasal drops of naphazoline, several solution-based formulations of xylometazoline were evaluated (xylometazoline alone and in combination with dexpanthenol or ipratropium), as well as solutions of oxymetazoline (0.025% and 0.05% *w/v* solution formulation applied as drops). As shown in Table 1, naphazoline has the lowest P_app_ among the tested sympathomimetics, regardless of the used cell model. The P_app_ values obtained in the much leakier RPMI 2650 model are significantly higher than the ones determined with the Calu-3 cell line. Nevertheless, both cell models reveal the same order of P_app_ values (P_app_ naphazoline < P_app_ oxymetazoline < P_app_ xylometazoline) and the drugs would be classified as having low permeability since the P_app_ values are lower than the P_app_ values of the high-permeability drug metoprolol (>1× 10^−5^ cm/s) [7,8]. As expected, there was no significant difference in the P_app_ values, meaning a concentration-independent permeability for oxymetazoline has been obtained for the tested formulations (nasal drops) that contain the same excipients but differ in the concentration of oxymetazoline. A similar observation can be made for the 10-fold dilutions of oxymetazoline drops, as well as for the nasal sprays containing combinations of xylometazoline with dexpanthenol. However, the P_app_ values for xylometazoline significantly differed for the evaluated formulations that contain only xylometazoline compared to its combination with another drug (dexpanthenol or ipratropium), with higher permeability of xylometazoline observed for the xylometazoline-only nasal formulation. Notably, the xylometazoline formulations differ in their osmolarity, with the osmolarity measurements demonstrating that the nasal sprays containing combination of xylometazoline with dexpanthenol are hypertonic (Table S4). It is a common notion that hypertonic solutions can increase the drug permeability. Moreover, although the primary effect of hyperosmotic solutions on mammalian cells is cell shrinking, the hyperosmolarity can, but does not necessarily, increase the paracellular drug permeability across a cell layer. As evident from Table 1, the observed permeability of xylometazoline from the hyperosmotic formulation of xylometazoline and dexpanthenol (Septanazal^®^ spray) is rather decreased compared to the isotonic formulations containing only xylometazoline, contrary to the anticipated increased drug permeability due to hyperosmolarity. As xylometazoline is lipophilic, it is very probable that this drug does not utilize the paracellular pathway to any relevant extent because it is absorbed primarily by passive diffusion. Lower P_app_ values of xylometazoline have also been obtained for the Otrivin^®^ Duo spray containing combination of xylometazoline and ipratropium, compared to the xylometazoline-only formulation. It would be interesting to see whether these in vitro observed differences in the permeability of xylometazoline are also mirrored in the pharmacokinetic parameters for these formulations. However, to our knowledge, no data comparing the pharmacokinetics of these different formulations containing xylometazoline are available. Minimal systemic absorption is expected following correct topical application of nasal spray containing xylometazoline, due to drug-induced vasoconstriction [33]. The results demonstrating even lower permeability of xylometazoline when combined with other drugs may indicate lower potential for systemic absorption; thus, no change in the systemic safety profile of the combination formulations is expected. This still shows the sensitivity of the cell models to a possible pharmacokinetic drug–drug interaction, and in case opposite results in terms of enhanced xylometazoline permeability were detected for a newly developed formulation, caution prior to in vivo testing could be advised.

Solution-based formulations of the antihistaminic drug azelastine (0.1% and 0.15%*w/v*), and fixed-dose combination nasal sprays containing fluticasone propionate as suspension and azelastine in solution form were also investigated, with the calculated P_app_ values presented in Table 1. Most notably, the azelastine P_app_ could not be determined for undiluted formulations due to the noticed substantial damage of the cell layers in both cell models. Namely, the damage to the cell layers could be observed by visual inspection, as some parts of the cell layers were detached from the insert membrane. The LDH cytotoxicity assay was used to determine the cell viability, as this was another criterion taken into account when deciding which results from the permeability assays can be included into calculating the mean P_app_ or flux values, if the criteria for adequate TEER values and P_app_ of LY at the end of the permeability assays could not be satisfied. Cell toxicity was not confirmed by the LDH assay (0% cytotoxicity), as the obtained absorbance values were comparable to the spontaneous LDH activity of the cell lines; however, the determined P_app_ values of LY were above the set criteria, and an evident drop of TEER was noticed at the end of the permeability assays. On the other hand, such an effect on the cell layer integrity was not noticed for the 10-fold diluted formulations (diluted with an isotonic assay buffer), which further confirms the usefulness of this experimental setup, besides the dilution factor being previously reported to be clinically relevant. We assume that any evaluation of nasal formulations using cell line models would benefit from including such dilution of the formulations investigated in the studies, especially in terms of achieving proper osmolarity prior to permeability assays and avoiding any potential problems with the cell layer integrity. Most of the tested marketed nasal formulations are isotonic, with the exception of the hypertonic nasal sprays containing sumatriptan, zolmitriptan, fixed-dose combination of xylometazoline with dexpanthenol, and the hypotonic nasal formulation of ciclesonide (Table S4). The osmolarity measurements showed that 10-fold dilution of the hyper- and hypotonic nasal formulations with an isotonic assay buffer resulted in obtaining isotonic formulations for permeability assessment. Despite the slight differences in the composition of the solution-based nasal formulations containing only azelastine as the active pharmaceutical ingredient (Table S1) at different concentrations, the calculated P_app_ values do not differ significantly (Table 1), and similar P_app_ values are obtained with both cell models, indicating concentration-independent permeability of azelastine. However, an almost twofold decrease in the P_app_ value of azelastine can be noticed for the nasal formulation containing a combination of azelastine and fluticasone propionate (Dymista^®^) (Table 1). Although azelastine is soluble in this formulation, the presence of suspended micronized fluticasone propionate particles requires the use of stabilizers for suspension formulations, such as microcrystalline cellulose and sodium carboxymethylcellulose. These two excipients create a hydrogel with a 3D network structure, supported by hydrogen bonding and ionic interactions among the microcrystalline cellulose, sodium carboxymethylcellulose, and water [34]. We believe that the reason for the twofold lower P_app_ value of azelastine from the suspension-based combination product Dymista^®^ is the complex network structure of the hydrogel limiting convection processes within it, thus extending the diffusion path of azelastine. The environment in the solution-based sprays of azelastine that contain hydroxypropylmethylcellulose does not hinder the diffusion of the dissolved azelastine to the same extent, as it can be reflected through the viscosity of the nasal sprays, which can significantly differ based on the excipients used and their concentration (microcrystalline cellulose and sodium carboxymethylcellulose, or hydroxypropylmethylcellulose) [35]. However, further studies focused on such in-depth characterization of the nasal sprays in terms of reverse engineering, determination of the quantitative composition, and investigation of the viscosity of the formulations at different concentrations of the used excipients, and assessment of other important properties of the nasal formulations are needed, as this was beyond the scope of our current study. Instead, we considered the possibility whether the observed twofold difference in the P_app_ values can be reflected in vivo. The plasma concentration profiles of azelastine from a combination product (azelastine + fluticasone propionate) and from a nasal formulation containing only azelastine did not show any difference in the azelastine bioavailability, as the pharmacokinetic parameters (AUC and C_max_) were very similar [27]. Nevertheless, it is important to highlight that very high variation in the pharmacokinetic parameters has been reported in the study (CV~40–60%) [27]. The reason for such discrepancy between the in vitro data and the in vivo behavior is not yet known, but does indicate that the cell models are very sensitive when utilized in permeability evaluation of nasal formulations and sometimes even detect differences between formulations which do not always manifest through differences in in vivo bioavailability. Obviously, the permeability alone is not always the rate-limiting step of drug absorption for the nasal pathway. The in vivo absorption and bioavailability of intranasally administered drugs can be a function of a complex interplay of different physiological, as well as drug-, formulation-, and device-related factors. As such, the permeability measurements must be viewed in the wider context of other relevant factors, such as spray pattern and plume geometry, which are important for the formulation deposition in the nose.

The permeability of azelastine from a solution in assay buffer with 1% MeOH was also tested across the cell models (Table 1). Significantly lower (*p* < 0.01) P_app_ of azelastine from the prepared solution was obtained with the RPMI 2650 model, compared to the P_app_ from the nasal formulations containing only azelastine, while no significant difference could be observed between the P_app_ of azelastine from the prepared solution and from the azelastine-only nasal formulations, when the Calu-3 model was used. With the Calu-3 cell line, the permeability of azelastine from the solution in assay buffer with 1% MeOH and from the nasal formulations is also comparable with the previously published data on the permeability of the high permeability reference drug metoprolol, [7,8], indicating that azelastine is a highly permeable compound. This would also explain the similarity in the obtained P_app_ values for azelastine with both cell models, as this is the only drug among all tested compounds found in solution-based marketed nasal formulations (Table 1) for which comparable permeabilities were observed.

The permeability of ipratropium from prepared solution in assay buffer, as well as from a solution-based nasal spray containing a combination of ipratropium and xylometazoline was investigated across the two cell models. The solutions of ipratropium in assay buffer were prepared at two different concentrations (0.6 mg/mL and 3 mg/mL for permeability assays with the RPMI 2650 and Calu-3 cells, respectively, due to anticipating analytical challenges in determination of ipratropium content in the samples and a need for using an LC-MS/MS analytical method when assessing the permeability across the tight Calu-3 cell model). There is no significant difference between the determined P_app_ values for the undiluted tested solutions in the RPMI 2650 cell model, indicating that the presence of xylometazoline does not influence the permeability of ipratropium (Table 1). Moreover, identical P_app_ values of ipratropium were obtained for the undiluted and 10-fold diluted Otrivin duo nasal spray for the RPMI 2650 model (Table 1), whereas the P_app_ of this drug from the nasal spray was determined only for the undiluted formulation using the Calu-3 cell line. The obtained P_app_ values of ipratropium with the Calu-3 model differ when the prepared solution in assay buffer and the marketed formulation were tested. However, the P_app_ values are in the same order of magnitude (×10^−7^ cm/s) and are consistent with the reported permeability of ipratropium (1.6 × 10^−7^ cm/s) in a study by Panduga et al. (2017) [36]. The example of testing the permeability of ipratropium shows that sometimes, in the cases of low permeable drugs, the leaky A–L RPMI 2650 cell model offers an advantage for permeability testing in terms of using a more convenient and affordable LC-UV analytical method for analyzing the permeability samples instead of an LC-MS/MS due to significantly higher concentrations of analytes obtained in the acceptor compartment.

The anti-migraine drugs sumatriptan and zolmitriptan stand out in this study, as they are intended for nose-to-brain delivery and their rapid absorption through the nasal mucosa is highly desired. The permeability of these two drugs from commercially available hypertonic intranasal sprays was tested at 10-fold dilution of the formulations, and solutions of the respective drugs in assay buffer (pH 7.4) were prepared in-house and utilized in permeability assays. Higher P_app_ values of sumatriptan and zolmitriptan from the prepared solutions in assay buffer are obtained with both cell models (Table 1), compared to the respective P_app_ values from the diluted commercially available nasal formulations. We believe that the reason is the different pH values of the in-house prepared (pH 7.4) and the diluted marketed solutions (slightly increased pH values were measured for sumatriptan and zolmitriptan sprays after dilution, i.e., pH 6.4 and 5.3, respectively, compared to the undiluted sprays, which have pH around 5.5 and 5.0, as stated in the summary of product characteristics). In a study by Yu and Zeng (2007) [37], it was shown that zolmitriptan transport involved passive diffusion and active transporters, such as P-gp, Na^+^–H^+^ exchanger, and organic cation–H^+^ exchanger. Lower transport rate for zolmitriptan has been observed at lower pH (pH 6), compared to pH 7.4, as with an increase in pH, the unionized fraction of the drug also increases [37]. Sumatriptan is in the form of hemisulfate salt in the nasal spray formulation (pH 5.5) (Table S1); thus, it may utilize the paracellular route for transport across the cell layers. These results imply that the cell models are able to reveal the influence of formulations composition in terms of pH on the permeability of the active drug. Furthermore, the obtained P_app_ values for sumatriptan and zolmitriptan are higher with the leakier RPMI 2650 cell line [7,12] compared to the values determined using the Calu-3 model. The presence of high fractions of ionized drugs in the nasal sprays may be a possible explanation for these results and observed differences. The P_app_ values for the two drugs reported with the Caco-2 cells are lower than those obtained with the RPMI 2650 cell line, but higher than those reported with the Calu-3 model [37,38]. Nevertheless, all the determined P_app_ values indicate that these drugs are not highly permeable [29,30]. Most notably, the permeabilities of the investigated anti-migraine drugs do not differ in size order when compared to other drugs specifically intended for local therapy of the nasal mucosa instead of the systemic uptake. This further emphasizes the relevance of other factors (such as retention on the mucosal surface and metabolism) for systemic absorption through the nasal mucosa and perhaps the effectiveness of the nose-to-brain transfer, which should not be ignored even at relatively low in vitro permeability or in vivo bioavailability.

**Figure 1:**
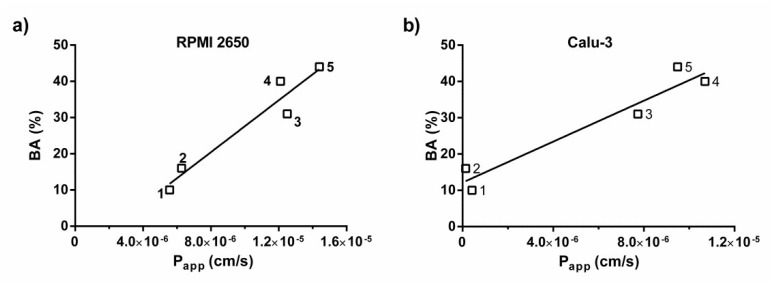
Correlation between P_app_ and systemic bioavailability of a limited number of intranasally administered drugs: 1- ipratropium, 2- sumatriptan, 3- budesonide, 4- azelastine and 5- triamcinolone acetonide in (**a**) the RPMI 2650 cell model; (**b**) the Calu-3 cell model.

Data on systemic availability of drugs administered intranasally in the literature is scarce, and the nasal bioavailability data for a limited number of drugs was retrieved from the published summaries of product characteristics for azelastine [39], sumatriptan [28], zolmitriptan [30], or reported literature data for ipratropium [31], triamcinolone acetonide [32] and budesonide [32,40]. The correlation between the experimentally determined P_app_ of the drugs administered intranasally as solutions and the nasal bioavailability data for a limited number of drugs (azelastine, ipratropium, sumatriptan, zolmitriptan) was evaluated by plotting values of P_app_ versus % bioavailability (Figure 1). Since the permeability of some drugs, such as azelastine and sumatriptan, could not be determined for the undiluted formulations due to observed cell layer damage or a limited amount of nasal sprays available for the permeability assays, the P_app_ values determined for the 10-fold diluted formulations of azelastine, sumatriptan and ipratropium were utilized in the correlation for the RPMI 2650 model, while for the Calu-3 model, the P_app_ values determined for the 10-fold diluted formulations of azelastine and sumatriptan, as well as the P_app_ value determined for the undiluted ipratropium nasal formulation, were used. Moreover, two intranasal corticosteroids were also included in the correlation analysis, by using the P_app_ values for the solutions of triamcinolone acetonide and budesonide in assay buffer with 1% DMSO. The correlation between the obtained P_app_ of the drugs administered intranasally as solutions and the nasal bioavailability data has been carried out for both the RPMI 2650 (Figure 1a) and Calu-3 (Figure 1b) cell models, excluding zolmitriptan from the correlation, due to being an outlier in both cell models. It is known that the anti-migraine drugs zolmitriptan and sumatriptan show bimodal absorption after nasal spray administration, indicating that gastrointestinal absorption also occurs [41,42]. The swallowed fraction of the drug undergoes first-pass metabolism, and both the absorption across the nasal mucosa and across the gastrointestinal tract contribute to the systemic exposure. We assume that despite zolmitriptan not being a highly permeable drug according to the calculated P_app_ values with both cell models, it has relatively high systemic bioavailability (42%) [29,30], as the oral bioavailability of the swallowed fraction after intranasal administration is relatively high.

### 3.2. Permeability of Drugs from Suspension-Based Nasal Formulations

Most of the intranasal corticosteroids are administered as suspension-based formulations, with the exception of betamethasone sodium phosphate, formulated as solution-based nasal drops, and triamcinolone acetonide, which, besides the suspension-based spray, also exists as a solution-based spray. The permeability of the first-generation corticosteroids triamcinolone acetonide and budesonide from suspension-based sprays was tested with the cell models as undiluted and as 10-fold diluted formulations. Moreover, 0.002% solutions of triamcinolone acetonide and budesonide in assay buffer containing 1% DMSO were prepared and the permeability of the respective drugs was tested with the two cell models. As triamcinolone acetonide also exists in solution-based nasal formulation (Tri-Nasal^®^) (0.05%), a solution with the same qualitative and quantitative composition as the Tri-Nasal^®^ formulation has been prepared in-house, according to [43] Hirsh and Tibbetts (2005), and the permeability was tested. The calculated mean flux and P_app_ values are reported in Table 2.

It can be noticed that for triamcinolone acetonide, significantly higher P_app_ values are obtained for the 1% DMSO in assay buffer solution compared to the in-house prepared solution having the same composition as the nasal formulation Tri-Nasal^®^. The obtained P_app_ values have one- to twofold higher orders of magnitude (comparison of 1% DMSO in assay buffer solution and in-house prepared nasal solution formulation), regardless of the cell model used. These results highlight the influence of the formulations composition on drug permeability which the tested cell models are able to detect. The permeability of budesonide from 1% DMSO solution in assay buffer is comparable to the permeability of triamcinolone, as the calculated P_app_ values are very similar, and the results obtained with the RPMI 2650 and Calu-3 cell models would suggest that both first-generation corticosteroids have permeability very close to that of the high permeability reference standard metoprolol [7,8].

Obtaining reproducible results when testing drug permeability from suspension-based formulations is quite challenging, even within the same laboratory, due to the following reasons: there can be a variation in the local drug concentration on the cells surface, as the drug particles may not be evenly distributed and areas having higher drug concentration can exist. Since the exact ratio between the dissolved and undissolved drug in the formulation on the donor side of the cells cannot be determined, and only the theoretical initial concentration based on the nominal drug amount and the applied volume can be considered, the P_app_ values of the drug cannot be calculated. Instead, only the flux values can be determined. Calculating the P_app_ values of drugs enables comparison between results obtained under somewhat different but still similar experimental settings and in different laboratories, although the inter-laboratory variability in terms of different cell culturing conditions may affect the results. Caution is required if there is a need to compare the flux of drugs from suspension-based formulations obtained in different laboratories, since the amount of drug permeated across the cell layers can differ if the initial nominal drug concentration applied is variable and the sink conditions are not maintained throughout the permeability assay. In this regard, it is important to keep in mind that the first-generation corticosteroids [44], such as triamcinolone acetonide and budesonide have higher aqueous solubility (21 μg/mL and 16 μg/mL, respectively) [45] than the first-generation corticosteroid beclomethasone dipropionate (0.13 μg/mL) [45] and the second-generation corticosteroids [44] (mometasone furoate (<0.1 μg/mL), fluticasone propionate (0.14 μg/mL), ciclesonide (<0.1 μg/mL)) [45,46]. Therefore, we opted to use an assay buffer with 4% BSA as medium in the permeability assays when testing formulations of beclomethasone dipropionate and the second-generation corticosteroids in order to maintain sink conditions.

Suspension-based nasal sprays containing the first-generation intranasal corticosteroid beclomethasone dipropionate and the second-generation corticosteroids fluticasone propionate, mometasone furoate and ciclesonide with low aqueous solubility were tested with the cell models and the calculated flux values for the evaluated formulations are shown in Table 3. A more notable decrease in the TEER values at the end of the permeability assays has been observed for the Calu-3 cell line, for both the undiluted and 10-fold diluted formulations, with the decrease in the TEER being more pronounced for the undiluted formulations, relative to the 10-fold diluted ones. For the RPMI 2650 cell line, a slight decrease or even increase in the TEER values was observed when testing these undiluted and 10-fold diluted suspension-based formulations. In this case, the interpretation of the obtained results for the TEER of the leaky RPMI 2650 cell model is quite challenging and a definite conclusion on whether or not the cell layer integrity has been maintained throughout the experiment, based solely on the measured TEER, cannot be drawn. This is a point where one should accept that TEER values on their own, unless distinctly altered by the experimental conditions, are simply not a reliable descriptor of the epithelial integrity for the very leaky epithelia such as the RPMI 2650. On the contrary, the TEER values can be well implemented, alongside with other parameters, as indicators of the cell layer integrity for the tight epithelia, such as Calu-3, and for the tight epithelial barriers, reasonable interpretation of the measured TEER can be made. Testing the permeability of low-molecular-weight paracellular markers has been reported to be a better indication of maintained cell barrier integrity than TEER values [47]; however, the permeability of LY across the cell models at the end of the permeability assessment of nasal formulations of these corticosteroids could not be performed, as mass balance studies were carried out. This inherently presents a drawback of the mass balance measurements and/or of the leaky epithelia, whose integrity during the experiments is more difficult to evaluate. Nevertheless, the results for the calculated flux values for the tested corticosteroid formulations presented in Table 3 were considered as being reliable, as the paracellular pathway is not very relevant for the lipophilic corticosteroids tested. Additionally, another important criterion that has to be taken into account for permeability calculations, i.e., the cell viability was investigated, where applicable, and minimal cytotoxicity was obtained with the LDH assay, with somewhat higher cytotoxicity observed when the RPMI 2650 model was used (4–10% cytotoxicity for undiluted formulations and 0–2% cytotoxicity for the 10-fold diluted ones). Furthermore, the most reliable criterion for the consistency of the model epithelial barrier, the linearity of the plot of the amount permeated to the acceptor side, has been satisfied.

It can be noticed that for the intranasal corticosteroids that have lower aqueous solubility than triamcinolone acetonide and budesonide, similar flux values are obtained for the undiluted and 10-fold diluted formulations, despite the different nominal concentrations of the drug. This implies that the in vitro models correctly capture the situation where the drug particles in nasal suspensions serve as a constant source of intranasally administered corticosteroids, allowing drug release for the duration of their residence on the nasal mucosa in the available fluid volume. Moreover, the flux values obtained with the two cell models do not differ significantly, except in the case of beclomethasone dipropionate when higher flux values are observed with the RPMI 2650 cell model (Table 3).

Testing the permeability of fluticasone propionate from three different formulations was of particular interest due to available literature data on their nasal bioavailability. Suspension-based spray (Flixonase^®^ spray) and nasal drops (Flixonase^®^ nasal drops) containing this drug as well as suspension-based spray containing combination of fluticasone propionate and azelastine (Dymista^®^) were compared in terms of the calculated flux values. As evident from Table 3, no difference in the flux values of the drug between the spray formulation containing only fluticasone propionate (Flixonase^®^) and the combination spray Dymista^®^ was observed with the RPMI 2650 model; however, 1.4- to 1.6-fold higher flux values are obtained with the Calu-3 cells. This implies that either the presence of azelastine in Dymista^®^, or the differences between the excipients comprising the formulations, can affect the in vitro permeability of fluticasone propionate through the Calu-3 cell layer. The differences in the flux values observed in vitro agree with the in vivo data, where a pharmacokinetic study shows lower systemic bioavailability of fluticasone propionate from a fluticasone propionate-only nasal spray (1.14%), compared to the 1.86% (fluticasone propionate+azelastine) [27]. With both cell lines, higher flux values of fluticasone propionate were obtained for the undiluted nasal drops, compared to undiluted Flixonase^®^ nasal spray, while no difference in the flux values of the drug between 10-fold diluted nasal drops and nasal spray formulation was observed.

Given that very similar flux values were obtained for the different formulations of fluticasone propionate, these flux values reflect the low aqueous solubility of the drug and indicate that equal concentrations of dissolved fluticasone propionate can be found in the formulations, despite the different nominal drug concentrations. Namely, the nominal drug concentration in the applied formulations are as follows: 1 mg/mL in the nasal drops, as declared by the manufacturer; 50 µg/actuation for Flixonase^®^ spray (147 µL volume per spray, as published in [46], resulting in nominal concentration of 0.34 mg/mL); 50 µg/137 µL Dymista^®^ spray, as declared by the manufacturer, i.e., nominal concentration of 0.37 mg/mL. The highest drug content is found in the applied volume of nasal drops; however, the nasal bioavailability of fluticasone propionate from this formulation is very low (0.06%) [32] and is strongly affected by the mucociliary clearance mechanism in vivo, the low aqueous solubility and slow dissolution rate of fluticasone propionate in the limited amount of nasal mucus [32], as well as the difference in the region of the nasal cavity where deposition of the nasal drops occurs (posterior ciliated region of the nose) [32,48], compared to the nasal spray (anterior non-ciliated part) [48]. Again, we can observe that the in vitro cell-based models for permeation assessment of nasal formulations can be discriminative, and the in vivo situation tends to reflect the permeability differences in the context of other relevant factors.

Both cell lines provide similar results for the flux of fluticasone propionate from the nasal drops formulation, with only the Calu-3 cell line being able to detect the differences between the nasal sprays containing only fluticasone propionate or fixed-dose combination with azelastine. Similar results were also obtained in a study with the 3D human bronchial tissue model EpiAirway™, when the absorption of fluticasone propionate from the combination nasal spray (fluticasone propionate+azelastine, Dymista^®^) and from nasal spray containing only fluticasone propionate (Flonase^®^) was tested [49]. Namely, after 4 h, higher permeation of fluticasone propionate has been reported from Dymista^®^ compared to Flonase^®^ spray. Currently, it is unclear why the difference in the flux of the drug from the Flixonase^®^ and Dymista^®^ sprays is not that much pronounced with the RPMI 2650 cells, as it is with the Calu-3 model. On the other hand, in our study, for all the other investigated drugs, similar results with the RPMI 2650 cells were demonstrated to the ones obtained with the Calu-3 cell line.

### 3.3. Applicability of the RPMI 2650 and Calu-3 Cell Models for Permeability Assessment of Nasal Formulations

The Calu-3 cell line has received more research attention than the RPMI 2650 cells, as different research groups have investigated the potential of the Calu-3 model to be used for drug permeability assessment, with a recent work of Inoue et al. [17] demonstrating a good correlation between the P_app_ values of a set of drugs obtained with the Calu-3 cell line and the nasal drug permeation rate, as well as an excellent correlation of P_app_ values across the Calu-3 model and the nasal bioavailability. Our work revealed that both the RPMI 2650 and Calu-3 cell models, although differing in the number of cell layers (monolayers observed in the Calu-3 model vs. multilayers in the RPMI 2650 cell line), the TEER (much lower TEER values in the RPMI 2650 model) and origin of the cells (nasal origin of RPMI 2650 vs. bronchial of Calu-3) [12,18] can be considered as being comparable in terms of offering information on drug permeability and revealing differences between nasal formulations. In general, the two cell models could show the influence of the pH, the presence of another drug and different excipients in the formulations on drugs permeability, as differences in the in vitro drug permeation could be demonstrated, and these in vitro observed differences, in few cases, also correlated with the differences in the in vivo bioavailability between the formulations. Earlier, we have also demonstrated that the RPMI 2650 and Calu-3 cell models can correctly predict the outcome of bioequivalence testing of nasal drug products containing first-generation corticosteroids [23]. Moreover, a previous study utilizing the RPMI 2650 cell line for showing the effect of different formulations on the permeability of ketoprofen has successfully demonstrated permeation-enhancing effect of hybrid alginate-pectin and alginate-carrageenan microparticles [19]. We therefore see the potential and a need for both cell models to continue being utilized in nasal formulation testing during formulation development and optimization. However, the understanding of the interplay of multiple factors influencing the nasal bioavailability besides the permeability, such as the mucociliary clearance limiting the contact time of the drug with the nasal mucosa [9], nasal deposition site [32] and formulation- and device-related factors (e.g., viscosity, spray pattern, plume geometry) [50], will have to be further improved. The use of each individual cell line, however, brings some challenges. As shown in our current work, utilizing only the measured TEER values of the leaky RPMI 2650 cell model to evaluate whether or not the cell layer integrity has been maintained is difficult and requires alternative methods for investigation of the cell barrier integrity. In this regard, the use of the Calu-3 cell model is an option for avoiding this challenge with results from the TEER measurements. When the permeability of low-permeable compounds is tested, the utilization of the Calu-3 cell model in permeability assays may require the use of an LC-MS/MS analytical method due to significantly lower concentrations of analytes obtained in the acceptor compartment. In such cases, using a leakier RPMI 2650 model allows the more affordable LC-UV analytical methods to be implemented for analysis of the permeability assay samples.

The available in vitro and in vivo models for nasal drug permeability assessment have different complexities, and none of them have a fully predictive potential for a precise estimation of the nasal drug bioavailability. The less complex models such as the cell lines can be utilized for investigating the drug transport mechanisms and estimation of the drug permeability, while the more complex ones provide information on the bioavailability and have a greater predictive potential. The in vitro cell models utilized in our study cannot completely simulate all the factors affecting the processes from deposition of the drug on the surface of the nasal mucosa until its absorption across the nasal epithelium (e.g., the mucociliary clearance, post-nasal drip, retention time of the drug in the nose, etc.), but do offer information on drugs permeability properties, which can complement the information gained when more complex models are used. Thus, regardless of the difference in their complexity, the cell lines as well as the more complex in vivo models are needed, as synergy of information is gained with their utilization necessary for the prediction of drug bioavailability.

## 4. Conclusions

The RPMI 2650 and Calu-3 cell models of the nasal epithelial barrier cultured at an A–L interface have the potential to detect the differences in drug permeability from nasal formulations and to reveal the effect of the formulation on drug permeability. The results from the permeability assays using the two cell models are comparable and both models are sufficiently discriminative, especially when used in tandem, where with the different minor weaknesses and advantages, the two models complement each other. They demonstrate differences in the in vitro drug permeation comparable to the in vivo bioavailability, which is also subject to other factors besides the drug permeability. The investigated cell lines can be applied for evaluation of in vitro permeation of intranasally administered drugs having local and systemic effect from solution- and suspension-based formulations.

## Figures and Tables

**Table 1:** Calculated P_app_ values for different intranasally administered drugs from solution-based formulations and in-house prepared solutions in assay buffer, 1% DMSO or 1% MeOH in assay buffer, when A–L RPMI 2650 and A–L Calu-3 models are utilized. Published data on systemic bioavailability is also presented.

Drug	Tested Formulation	Concentration (mg/mL)	A–L RPMI 2650 Model		A–L Calu-3 Model		Nasal Bioavailability (%)
			P_app_ ± SD (×10^−7^ cm/s)		P_app_ ± SD (×10^−7^ cm/s)		
			Undiluted Formulation	10-Fold Diluted Formulation	Undiluted Formulation	10-Fold Diluted Formulation	
Naphazoline	Benil^®^ drops	1	6.29 ± 0.29	3.58 ± 0.39	3.21 ± 0.24	2.51 ± 0.34	No data available
Oxymetazoline	Operil^®^ drops	0.25	52.9 ± 3.69	58.9 ± 1.67	5.85 ± 0.32	5.95 ± 0.37	No data available
	Operil^®^ drops	0.5	50.5 ± 3.99	49.4 ± 3.91	5.31 ± 0.57	5.96 ± 0.66	
Xylometazoline	Maresyl spray	1	74.0 ± 4.54	68.9 ± 2.49	23.0 ± 1.43	32.1 ± 3.32	No data available
Xylometazoline + Dexpanthenol	Septanazal^®^ spray	0.5 ^a^	53.2 ± 1.46	50.8 ± 1.94	12.3 ± 2.16	12.1 ± 1.78	No data available
	Septanazal^®^ spray	1 ^a^	53.6 ± 4.65	56.3 ± 4.36	9.35 ± 1.40	14.2 ± 2.45	
Xylometazoline + Ipratropium	Otrivin^®^ Duo spray	0.5 ^a^	48.8 ± 11.50	41.6 ± 5.40	17.2 ± 1.27	17.3 ± 0.81	No data available
Azelastine	Allergodil^®^ Akut	1	cell layer integrity not maintained	121 ± 11.3	cell layer integrity not maintained	106 ± 9.4	40 [27]
	Azelastine HCl Nasal Solution 0.15%, Perrigo	1.5		121 ± 24.0		107 ± 16.6	
	Azelastine HCl Nasal Solution 0.15%, Apotex Corp.	1.5		136 ± 14.7		102 ± 5.66	
	Solution in assay buffer with 1% MeOH	0.01 ^b^	80.1 ± 4.84	/	97.9 ± 16.6	/	
Azelastine +Fluticasone propionate	Dymista^®^ spray	1 ^a^	cell layer integrity not maintained	61.2 ± 5.84	cell layer integrity not maintained	60.3 ± 2.80	
Sumatriptan	Sumatriptan Sandoz Nasal spray 20 mg	200	NT *	62.7 ± 6.19	NT *	1.39 ± 0.28	16 [28]
	Solution in assay buffer	0.14 ^c^	107 ± 18.1	/	3.38 ± 1.12	/	
Zolmitriptan	Zomig^®^ 5 mg Nasal spray	50	NT *	15.9 ± 1.63	NT *	0.73 ± 0.17	42 [29,30]
	Solution in assay buffer with 1% DMSO	0.1	89.6 ± 4.09	/	2.77 ± 0.20	/	
Ipratropium	Solution in assay buffer	0.6/3 ^d^	50.62 ± 8.95	/	1.74 ± 0.73	/	10 [31]
Ipratropium + Xylometazoline	Otrivin^®^ Duo	0.6 ^a^	55.4 ± 13.44	55.7 ± 5.31	4.33 ± 0.29	NT *	No data available
Triamcinolone acetonide	Solution in assay buffer with 1% DMSO	0.02	143.9 ± 8.96	/	94.9 ± 5.03	/	44 [32]
Budesonide	Solution in assay buffer with 1% DMSO	0.02	124.8 ± 11.31	/	77.4 ± 22.3	/	31 [32]

* NT = not tested due to limited amount of nasal formulations available for permeability assays. ^a^ Concentration only of the first drug shown for the combination formulations. ^b^ Solution of azelastine hydrochloride. ^c^ Solution of sumatriptan succinate. ^d^ Ipratropium bromide solutions tested at concentrations of 0.6 mg/mL and 3 mg/mL with the RPMI 2650 and Calu-3 models, respectively.

**Table 2:** Calculated mean flux and apparent permeability coefficient (P_app_) values (*n* ≥ 3) of budesonide and triamcinolone acetonide from nasal solution- and suspension-based formulations and prepared solutions in 1% DMSO. SD, standard deviation.

Drug	Tested Formulation	A–L RPMI 2650 Model		A–L Calu-3 Model	
		Mean flux ± SD (×10^−2^ µg/cm^2^/h)	P_app_ ± SD (×10^−7^ cm/s)	Mean flux ± SD (×10^−2^ µg/cm^2^/h)	P_app_ ± SD (×10^−7^ cm/s)
Budesonide	Tafen^®^ (undiluted)	214 ± 37	NA *	166 ± 15	NA *
	Tafen^®^ (10-fold diluted)	191 ± 13	NA *	73 ± 0.09	NA *
	Assay buffer with 1% DMSO	68.5 ± 6.88	125 ± 11	40.0 ± 9.86	77.4 ± 22.3
Triamcinolone acetonide	Nasacort^®^ AQ (undiluted)	81 ± 7	NA *	100 ± 8	NA *
	Nasacort^®^ AQ (10-fold diluted)	66 ± 4	NA *	49 ± 9	NA *
	Tri-Nasal^®^ (undiluted)	60.3 ± 9.94	3.35 ± 0.55	45.2 ± 5.96	2.51 ± 0.33
	Assay buffer with 1% DMSO	105 ± 11	143 ±14.6	69.7 ± 3.7	94.9 ± 5.03

* NA = not applicable.

**Table 3:** Calculated mean flux values (*n* ≥ 3) of intranasal corticosteroids with low aqueous solubility from different nasal suspension-based spray formulations and nasal drops. SD, standard deviation.

Drug	Tested Formulation	A–L RPMI 2650 Model		A–L Calu-3 Model	
		Mean Flux ± SD (×10^−2^ µg/cm^2^/h)			
		Undiluted Formulation	10-Fold Diluted Formulation	Undiluted Formulation	10-Fold Diluted Formulation
Beclomethasone dipropionate	Beconase AQ^®^	0.77 ± 0.24	0.66 ± 0.25	0.28 ± 0.13	0.28 ± 0.09
Ciclesonide	Omnaris^®^	0.41 ± 0.16	0.63 ± 0.20	0.47 ± 0.07	0.61 ± 0.17
Fluticasone propionate	Flixonase^®^ nasal drops	1.57 ± 0.14	1.40 ± 0.27	1.82 ± 0.21	1.60 ± 0.34
	Flixonase^®^ nasal spray	1.21 ± 0.14	1.32 ± 0.07	1.25 ± 0.11	1.31 ± 0.22
Fluticasone propionate+Azelastine	Dymista^®^	1.29 ± 0.13	1.63 ± 0.32	1.73 ± 0.13	2.07 ± 0.08
Mometasone furoate	Mommox^®^	0.78 ± 0.37	0.87 ± 0.22	0.91 ± 0.12	1.15 ± 0.20

## Data Availability

Not applicable.

## Supplementary Materials

The following supporting information can be downloaded at: https://www.mdpi.com/article/10.3390/pharmaceutics14020369/s1, Table S1: Qualitative composition of the tested nasal formulations, Table S2: Summary of UHPLC experimental conditions, Table S3: MRM acquisition data used to quantify the presented analytes by LC-MS/MS, Table S4: Osmolarity of the tested undiluted and 10-fold diluted nasal formulations.
