# Supplementary material for: Applicability of RPMI 2650 and Calu-3 Cell Models for Evaluation of Nasal Formulations
Source: Pharmaceutics. 2022 Feb 6;14(2):369. doi: 10.3390/pharmaceutics14020369 (PMC8877043; doi:10.3390/pharmaceutics14020369)
Supplement: Supplementary file 1 [file pharmaceutics-14-00369-s001.zip › pharmaceutics-1556804-supplementary.pdf]

pharmaceutics

MDPI

Supplementary Materials: Applicability of RPMI 2650 and Calu-3 Cell Models for Evaluation of Nasal Formulations

Nadica Sibinovska, Simon Žakelj, Jurij Trontelj and Katja Kristan

Table S1. Qualitative composition of the tested nasal formulations.

Drug	Tested formulation (Manufacturer)	Excipients*
Naphazoline nitrate	Benil® drops 1mg/ml (Krka, Slovenia)	Boric acid, sodium tetraborate, tiomersal, glycerol, purified water
Oxymetazoline HCl	Operil® drops 0.25 mg/ml (Lek Pharmaceuticals, Slovenia)	Benzalkonium chloride, sodium hydroxide, sodium dihydrogen phosphate dihydrate, sodium hydrogen phosphate dihydrate, water for injection
	Operil® drops 0.5 mg/ml (Lek Pharmaceuticals, Slovenia)	
Xylometazoline HCl	Maresyl spray 1 mg/ml (Jadran Galenski Laboratorij, Croatia)	Purified saline water, potassium dihydrogen phosphate, purified water
Xylometazoline HCl +Dexpanthenol	Septanazal® spray 0.5 mg/ml (Krka, Slovenia)	Potassium dihydrogen phosphate, sodium monohydrogen phosphate dodecahydrate, purified water
	Septanazal® spray 1 mg/ml (Krka, Slovenia)	
Xylometazoline HCl + Ipratropium Br	Otrivin® Duo spray (GlaxoSmithKline, United Kingdom)	Disodium edetate, glycerol (85%), hydrochloric acid, sodium hydroxide, purified water
Azelastine HCl	Allergodil® Akut 1 mg/ml (MEDA Pharma GmbH & Co.KG, Germany)	Sodium edetate, hypromellose, sodium monohydrogen phosphate dodecahydrate, citric acid, sodium chloride, purified water
	Azelastine HCl Nasal Solution (Nasal Spray), 0.15% (Perrigo, Israel)	Sorbitol, sucralose, hypromellose, sodium citrate, edetate disodium, benzalkonium chloride, purified water (pH 6.4)
	Azelastine Hydrochloride Nasal Spray 0.15% (Apotex Inc., Canada)	Benzalkonium chloride, citric acid, dibasic sodium phosphate, edetate disodium, hypromellose, purified water, sodium chloride
Azelastine HCl+Fluticasone propionate	Dymista® nasal spray (MEDA Pharma GmbH & Co.KG, Germany)	Glycerin, microcrystalline cellulose, carboxymethylcellulose sodium, phenylethyl alcohol, edetate disodium, benzalkonium chloride, polysorbate 80, purified water
Sumatriptan	Sumatriptan Sandoz nasal spray 20 mg (Sandoz, Germany)	Potassium dihydrogen phosphate, dibasic sodium phosphate anhydrous, sulphuric acid, sodium hydroxide, purified water
Zolmitriptan	Zomig® 5 mg nasal spray (AstraZeneca, United Kingdom)	Citric acid, disodium phosphate, purified water
Triamcinolone acetonide	Nasacort® AQ nasal spray (Sanofi-Aventis U.S LLC, NJ, USA)	Microcrystalline cellulose, carboxymethylcellulose sodium, polysorbate 80, dextrose, benzalkonium chloride, edetate disodium, hydrochloric acid or sodium hydroxide, purified water
	Solution prepared in-house, according to Hirsh and Tibbetts [40]	Propylene glycol, polyethylene glycol 3350, edetate disodium, citric acid, sodium citrate, benzalkonium chloride, purified water
Budesonide	Tafen® (Lek Pharmaceuticals, Slovenia)	Dispersible cellulose (microcrystalline cellulose and carboxymethylcellulose sodium, (89:11, w/w)), polysorbate 80, potassium sorbate E 202, glucose anhydrous, disodium edetate, hydrochloric acid concentrated, ascorbic acid E 300, purified water
Beclomethasone dipropionate	Beconase AQ® (GlaxoSmithKline, United Kingdom)	Avicel RC 591 (Microcrystalline cellulose and carboxymethylcellulose sodium), glucose anhydrous,

		polysorbate 80, benzalkonium chloride, phenylethyl alcohol, purified water
Fluticasone propionate	Flixonase® nasal drops (GlaxoSmithKline, United Kingdom)	Polysorbate 20, sorbitan laurate, sodium dihydrogen phosphate dihydrate, disodium phosphate anhydrous, sodium chloride, water for injections
	Flixonase® spray (GlaxoSmithKline, United Kingdom)	Dextrose anhydrous, microcrystalline cellulose and carboxymethylcellulose sodium (Avicel RC591), phenylethyl alcohol, benzalkonium chloride, polysorbate 80, dilute hydrochloric acid, purified water
Ciclesonide	Omnaris® (AstraZeneca, Canada)	Microcrystalline cellulose, carboxymethylcellulose sodium, hypromellose, potassium sorbate, edetate sodium, hydrochloric acid, purified water
Mometasone furoate	Mommox® (Lek Pharmaceuticals, Slovenia)	Microcrystalline cellulose (E460), Sodium croscarmellose (E468), glycerol (E442), citric acid monohydrate (E330), sodium citrate dihydrate (E331), polysorbate 80 (E433), benzalkonium chloride, water for injection

* as described in the respective Summary of product characteristics or Patient information leaflet

Table S2. Summary of UHPLC experimental conditions.

Analyte	Mobile Phase	Column	Detection Wavelength (nm)	Flow Rate (mL/min)	Retention time (min)
Azelastine hydrochloride	Ammonium phosphate buffer (pH = 3.0):ACN = 65:35	Acquity HSS C18 1.7 µm, 50 × 2.1 mm	220	0.5	1.2
Budesonide	Sodium phosphate buffer (pH = 3.2):ACN = 70:30	Acquity BEH C18 1.7 µm, 50 × 2.1 mm	254	0.8	Epimer A: 2.3<br>Epimer B: 2.5
Ipratropium bromide*	MFA = MilliQ H_2O:ACN:CF_3COOH= 900:100:1<br>MFB = MilliQ H_2O:ACN<br>MFA:MFB = 85:15	Acquity BEH C18 1.7 µm, 100 × 2.1 mm	253	0.3	1.9
Naphazoline nitrate	Phosphate buffer (pH = 2.8):MeOH = 70:30	Acquity BEH C18 1.7 µm, 50 × 2.1 mm	280	0.3	2.2
Oxymetazoline hydrochloride	Ammonium phosphate buffer (pH = 3.0):ACN = 70:30	Kinetex C18 100A 2.6 µm, 50 × 2.1 mm	220	0.5	0.45
Sumatriptan	NaOH-$NaHCO_3$ buffer (pH 10.5):ACN = 75:25	Acquity BEH Phenyl 1.7 µm, 100 × 2.1 mm	227	0.4	2.2
Triamcinolone acetonide	MilliQ H_2O: ACN = 35:65	Acquity HSS C18 1.7 µm, 50 × 2.1 mm	254	0.3	0.55
Xylometazoline hydrochloride	Ammonium phosphate buffer (pH = 3.0):ACN = 70:30	Kinetex C18 100A 2.6 µm, 50 × 2.1 mm	220	0.5	0.9
Xylometazoline hydrochloride (+ dexpanthenol)	A- ammonium phosphate buffer (pH = 3.0); B- ACN<br>t (min) A% B%<br>initial 95 5<br>0.5 95 5<br>2.5 40 60<br>3.0 40 60<br>3.7 95 5<br>4.0 95 5	Kinetex C18 100A 2.6 µm, 50 × 2.1 mm	220	0.4	0.8
Zolmitriptan	NaOH-$NaHCO_3$ buffer (pH 10.5):ACN = 75:25	Acquity BEH Phenyl 1.7 µm, 100 × 2.1 mm	285	0.4	2.3

*method used for analysis of samples obtained in permeability assays with the RPMI 2650 cell model

Table S3. MRM acquisition data used to quantify the presented analytes by LC-MS/MS.

Analyte	MRM *m/z* transition<br>Precursor → product(qualifier)	Fragmentor (V)	Collision energy (eV)
Beclomethasone dipropionate	521.2 → 503.3 (57.2)	126	1 (37)
Ciclesonide	541.3 → 523.4 (323.2)	126	1 (9)
Fluticasone propionate	501.2 → 313.2 (293.2)	78	5 (9)
Ipratropium	332.1 → 166.2 (124.2)	110	37 (41)
Mometasone furoate	521.2 → 355.2 (147.1)	78	9 (21)

Table S4. Osmolarity of the tested undiluted and 10-fold diluted nasal formulations.

Drug	Tested formulation	Osmolarity (mOsm/kg)<br>Undiluted formulation	 10-fold diluted formulation
**Naphazoline**	Benil® drops	270	282
Oxymetazoline	Operil® drops 0.25 mg/ml	304	283
	Operil® drops 0.5 mg/ml	323	288
Xylometazoline	Maresyl spray	287	285
Xylometazoline +Dexpanthenol	Septanazal® spray 0.5 mg/ml	425	291
	Septanazal® spray 1 mg/ml	424	297
Xylometazoline + Ipratropium	Otrivin® Duo spray	275	283
Azelastine	Allergodil® Akut	296	285
	Azelastine HCl Nasal Solution 0.15%, Perrigo	315	286
	Azelastine HCl Nasal Solution 0.15%, Apotex Corp.	330	290
Azelastine +Fluticasone propionate	Dymista® spray	295	288
Sumatriptan	Sumatriptan Sandoz Nasal spray 20 mg	792	345
Zolmitriptan	Zomig® 5 mg Nasal spray	419	300
Triamcinolone acetonide	Nasacort® AQ	344	288
Budesonide	Tafen®	323	286
Beclomethasone dipropionate	Beconase AQ®	330	288
Fluticasone propionate	Flixonase® nasal drops	283	282
	Flixonase® spray	337	301
Ciclesonide	Omnaris®	30	261
Mometasone furoate	Mommox®	294	282
